# Antibody-Mediated Rejection and Sponge Effect in a Redo Lung Transplant Recipient

**DOI:** 10.1155/2021/6637154

**Published:** 2021-06-10

**Authors:** Ashwini Arjuna, Michael T. Olson, Sofya Tokman, Rajat Walia, Thalachallour Mohanakumar, A. Samad Hashimi, Michael A. Smith, Ross M. Bremner, Ashraf Omar

**Affiliations:** ^1^Norton Thoracic Institute, St. Joseph's Hospital and Medical Center, 500 W. Thomas Road, Ste. 500; Phoenix, AZ 85013, USA; ^2^University of Arizona College of Medicine-Phoenix Campus, 475 N 5th St, Phoenix, AZ 85004, USA; ^3^Norton Thoracic Institute Research Laboratory, St. Joseph's Hospital and Medical Center, 500 W. Thomas Road, Ste. 500; Phoenix, AZ 85013, USA

## Abstract

Long-term survival after lung transplant remains severely limited by chronic lung allograft dysfunction. Antibody-mediated rejection of lung transplant allografts is usually caused by donor-specific antibodies (DSAs) directed toward donor human leukocyte antigens (HLAs). Typically, patients with antibody-mediated rejection have significantly higher circulating DSAs and increased mean fluorescence intensity than those without antibody-mediated rejection. However, some patients with antibody-mediated rejection have low mean fluorescence intensities, partly due to the “sponge effect” related to DSAs binding to HLA molecules within the lung. Herein, we report the case of an 18-year-old, female lung transplant recipient who required retransplantation and developed circulating DSAs directed toward the first allograft but detected in circulation only after retransplantation. The present case draws attention to a rare finding of sponge effect in a patient with antibody-mediated rejection leading to allograft failure.

## 1. Introduction

Lung transplantation is a life-saving therapy for some patients with end-stage lung disease; however, survival after lung transplant is shorter than survival after other solid organ transplants. Chronic lung allograft dysfunction (CLAD) is the leading cause of death among lung transplant recipients who survive beyond the first year of transplant, and CLAD is driven by a variety of immune and nonimmune mechanisms [1]. Antibody-mediated rejection (AMR) is increasingly recognized as a risk factor for CLAD development and allograft failure [2–5]. Key diagnostic criteria for AMR include the presence of donor-specific antibodies (DSAs) directed toward donor human leukocyte antigens (HLAs) and characteristic lung histology with or without evidence of complement 4d (C4d) deposition within the allograft [3]. Typically, patients with AMR have significantly higher circulating DSA titers and increased mean fluorescence intensity (MFI), a surrogate marker for antibody titers, than those without AMR [6]. However, some patients with AMR have scant DSAs in circulation partly due to the “sponge effect” related to DSAs binding to HLA molecules within the lung. DSA adsorption in the graft was first described decades ago [7]; more recently, Visentin et al. [8] found that DSAs within the graft impacted posttransplant survival. The sponge effect was also described earlier by Girnita et al. [9], who reported a case of a lung transplant recipient who developed circulating DSAs against the first allograft after lung retransplantation. Herein, we report a case of an 18-year-old, female bilateral lung transplant recipient who underwent redo lung transplantation and subsequently developed circulating DSAs directed against the first allograft. The purpose of this case report is to highlight the unusual phenomenon of sponge effect in a patient with CLAD caused by AMR requiring retransplantation.

## 2. Case Report

An 18-year-old, female patient with a history of end-stage cystic fibrosis underwent bilateral sequential lung transplant with no major intraoperative complications in May 2017. Retrospective crossmatch results were negative for both donor T and B lymphocytes. Cytomegalovirus (CMV) status was positive in both the donor and the recipient. Early postoperative complications included a left foot drop and vocal cord palsy, which were treated with extensive physical therapy and a left vocal cord injection. One-month surveillance bronchoscopy with transbronchial biopsy and bronchoalveolar lavage (BAL) demonstrated no evidence of acute cellular rejection, and circulating DSAs were not detected.

**Figure 1:**
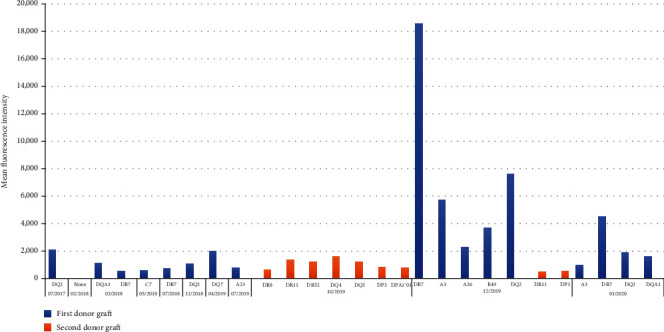
Mean fluorescence intensity of posttransplant donor-specific antibodies to donor human leukocyte antigens.

In July 2017, follow-up monitoring revealed the development of *de novo* DSA (DQ2 2,082 MFI). The patient received 1 dose of intravenous immunoglobulin (IVIG) (200 mg/kg) and subsequently developed signs and symptoms of aseptic meningitis. Her immunosuppressive regimen was optimized by increasing the dose of mycophenolate mofetil (500 mg BID to 750 mg BID), which reduced DSA (<1,000 MFI); allograft function at this time was normal (forced expiratory volume in one second [FEV_1_] 2.14 L, 85% predicted). She continued to have serial DSA analysis (Figure 1), which showed mainly class II DSAs with low MFIs. She continued to receive IVIG infusions (200 mg/kg) monthly and maintained good allograft function.

In December 2018, 19 months after transplant, she was admitted to the hospital with lung parenchymal ground-glass opacities on CT scan of the chest and acute hypoxemic respiratory failure. Circulating DSAs were detected but had a low MFI (DQ2 1,082 MFI). The patient was treated with intravenous corticosteroids (3 doses, 250 mg/kg) and, subsequently, antithymocyte globulin (ATG) (1.5 mg/kg, as tolerated, 3 doses). Although the decline in lung function temporarily plateaued after ATG, the patient was readmitted 2 months later with worsening hypoxemia and hypercapnia, eventually requiring veno-venous extracorporeal membrane oxygenation support and redo bilateral lung transplant 23 months after her first transplant.

The bilateral lung redo transplant was complicated by intraoperative hemorrhage requiring massive transfusion (22 units of packed red blood cells, 26 units of platelets, and 20 units of cryoprecipitate) and grade 3 primary graft dysfunction. Virtual and retrospective cross-match results were negative for T and B lymphocytes; however, circulating DSAs toward DQ7 (2000 MFI) were detected. The patient required prolonged mechanical ventilation and extensive rehabilitation before discharge two months after redo transplant.

**Figure 2:**
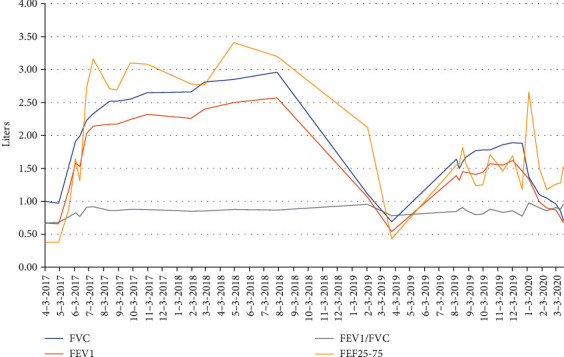
Posttransplant pulmonary function. FEV1: forced expiratory volume in one second; FEV1/FVC: forced expiratory volume in one second/forced vital capacity ratio; FEF25-75: mid-expiratory flow rate; FVC: forced vital capacity.

In July 2019, circulating DSAs against the HLA of the first donor antigens, HLA A23 (785 MFI), were detected in the setting of reduced lung function (FEV_1_ 1.39 L, 55% predicted). In October 2019, follow-up monitoring revealed circulating DSAs to several HLA class II antigens of the second donor, including DR8 (642 MFI), DR11 (1,380 MFI), DR52 (1,218 MFI), DQ4 (1,629 MFI), DQ5 (1,221 MFI), DP3 (809 MFI), and DPA1^∗^01 (780 MFI). The patient was treated with plasmapheresis (1.5 plasma volume with half fresh-frozen plasma and half albumin, 5 rounds). From July 2019 to December 2019, her pulmonary function gradually improved from 1.39 L to a peak of 1.62 L (64% predicted); her chest CT scan was unremarkable.  Figure 2 illustrates the patient's pulmonary function after both her primary and redo transplant.

In January 2020, the patient presented with dyspnea, a decline in lung function, and new CT chest findings of bilateral consolidations with scattered noncalcified nodules. She was hospitalized, had a bronchoscopy with transbronchial biopsy and BAL that demonstrated no evidence of acute cellular rejection or infection, and had a repeat serum DSA analysis that showed persistent DSAs to the second donor lung (DQ4 [1,296 MFI], DR11 [505 MFI], and DP3 [557 MFI]). In addition to DSAs against the second donor, she also had DSAs directed against the following mismatched antigens of the first donor allograft: DR7 (18,553 MFI), A3 (5,740 MFI), A26 (2,299 MFI), B49 (3,672 MFI), and DQ2 (7,621 MFI). Given her symptoms, changes on chest CT, and need for supplemental oxygen, she was treated for probable AMR with plasmapheresis, IVIG (200 mg/kg), and ATG (1.5 mg/kg, as tolerated, 3 doses). The patient gradually improved over the following week and was discharged home on room air. She continued outpatient plasmapheresis and IVIG for CLAD caused by AMR (restrictive allograft syndrome [RAS] phenotype). Despite these aggressive interventions, the patient developed a progressively worsening restrictive ventilatory defect and hypoxemia, which ultimately led to her transition to hospice and untimely death.

## 3. Discussion

AMR is a known cause of allograft failure after lung transplant and is thought to be driven by DSAs, which are often found in circulation. The sponge effect poses a unique challenge to diagnosing AMR, as circulating DSAs may be absent, or the MFI may be low. Diagnosing AMR early is important, as it allows for early intervention which potentially reduces allograft dysfunction and prolongs patient survival. In addition, identifying circulating DSAs may be important for donor selection in the case of retransplant to avoid a recurrent HLA-mismatch. This case suggests that the first allograft failed due to AMR with sponge effect, as explanting the first allograft led to a steep rise in circulating DSAs against the first donor lung. We propose that the second transplant met the same fate.

In a sentinel study conducted by Visentin et al. [8], class I and II anti-HLA antibody single-antigen flow bead assays were performed in 53 lung transplant recipients to identify intragraft DSAs in biopsy specimen eluates and in sera. Twenty-eight (52.8%) lung transplant recipients had serum DSAs, and 11 (20.8%) had intragraft DSAs. One-year postbiopsy graft survival was significantly lower for recipients with intragraft DSAs (*P* = 0.008, log-rank test). Thus, the authors concluded that intragraft DSAs may be an important marker for recipients with a higher risk of allograft loss.

Girnita et al. [9] were the first to describe a lung retransplantation case wherein DSA was detected in serum only after explantation of the first allograft that was suspected of having AMR. Once the patient received a second lung transplant, circulating DSAs directed toward the previous allograft were detected. Similarly, in our patient, circulating DSAs directed toward the first donor were mainly undetectable or had low MFI prior to redo transplant, and the MFI rose steeply after retransplantation. This raises the possibility that the patient died of AMR in spite of virtually undetectable circulating DSAs, as the DSAs were bound to the donor's lungs and therefore removed from circulation (i.e., sponge effect). Because DSAs against the first donor were detected following retransplantation and remained significantly elevated in the context of allograft injury, we propose that they may have contributed to AMR after retransplantation, even though they were not directed toward the second donor (possibly due to cross-reactive epitopes). Future studies are warranted to understand the implications of antibodies to previously failed allografts in the pathogenesis of AMR following retransplantation.

This study has certain limitations. One, the body of evidence collected herein represents the findings from only one redo lung transplant recipient with AMR against both the first and second donor allografts. Two, once the allograft is removed, antibodies bound to the graft can be identified by elution studies. However, the elution of antibodies from either of the allografts was not performed at our center. After retransplant, elution of antibodies bound to the second allograft and their characterization was not performed; therefore, it is uncertain whether there was cross-reactivity between circulating DSAs against the first donor and the second allograft. Lastly, C4d staining was absent in the second allograft, which may further demonstrate the limitations in defining AMR by the presence or absence of C4d staining.

This case draws attention to sponge effect, a rare manifestation of AMR following lung retransplantation. We conclude that the absence of circulating DSAs does not rule out AMR. We also postulate that circulating DSAs directed toward a first allograft may drive AMR after retransplant due to cross-reactive epitopes.

## Data Availability

This is a case report. References from PUBMED are included in the manuscript.

## Abbreviations

AMR: Antibody-mediated rejection
ATG: Antithymocyte globulin
BAL: Bronchoalveolar lavage
C4d: Complement 4d
CLAD: Chronic lung allograft dysfunction
CMV: Cytomegalovirus
DSA: Donor-specific antibody
FEV_1_: Forced expiratory volume in one second
HLA: Human leukocyte antigen
IVIG: Intravenous immunoglobulin
MFI: Mean fluorescence intensity
RAS: Restrictive allograft syndrome.

## Additional Points

This case study was presented at the 2021 American Transplant Congress held virtually, June 4-9, 2021.
